# Choroidal thickness in obese women

**DOI:** 10.1186/s12886-016-0227-z

**Published:** 2016-05-04

**Authors:** Erhan Yumusak, Kemal Ornek, Senay Arıkan Durmaz, Aydin Cifci, Hatice Ayhan Guler, Zehra Bacanli

**Affiliations:** Department of Ophthalmology, Kırıkkale University, School of Medicine, Kırıkkale, Turkey; Department of Endocrinology, Kırıkkale University, School of Medicine, Kırıkkale, Turkey; Department of Internal Medicine, Kırıkkale University School of Medicine, Kırıkkale, Turkey

**Keywords:** Choroidal thickness, Obese women, Optical coherence tomography

## Abstract

**Background:**

Excessive weight is a well-known risk factor for microvascular diseases. Changes in thickness in a vascular tissue, such as the choroid, can be useful to evaluate the effect of obesity on the microvascular system. The aim of this study was to evaluate the choroidal thickness (CT) changes in obese women, using optical coherence tomography (OCT).

**Methods:**

The prospective clinical study included examination of the right eyes of 72 patients. The right eyes of 68 patients were examined and served as the controls. A complete ophthalmological examination and OCT imaging were performed for each group studied. The CT in each eye was measured using OCT.

**Results:**

The obese group consisted of 72 female patients with a mean age of 37.27 ± 1.18 years. The control group included 68 female subjects with a mean age of 37.85 ± 7.98 years (*p* > 0.05). There was no statistical significant difference for the foveal retinal thickness measurements between the two groups (*p* > 0.5). Our study revealed significant choroidal tissue thickening subfoveally and at areas 500 **μ**m temporal, 500 μm nasal, and 1500 μm nasal to the fovea in the obese group (all *p* < 0.05). There was a positive correlation between body mass index (BMI) and CT changes.

**Conclusions:**

CT may increase in obese women and a positive correlation was found between BMI and CT.

The trial protocol was approved by the Local Ethical Committee of the Kırıkkale University, date of registration: April 27, 2015 (registration number: 10/11).

## Background

Obesity is a common health problem and its prevalence is increasing worldwide [1–3]. Excessive weight is a well-known risk factor for diabetes, hypertension, dyslipidemia, and microvascular diseases [4–6], including retinal vasculature [7, 8]. One of the main concerns with obesity is that microvascular alterations cannot be diagnosed in the early stages. Although many studies have investigated the comorbidities associated with obesity [9–11], predicting the risk of developing vascular damage remains challenging.

The association of obesity with cataract formation, glaucoma, and age-related macular degeneration has been shown in varying degrees. Researchers have hypothesized that retinal microvascular changes are precursors to developing obesity based on experimental and clinical observations [12, 13]. In the Blue Mountains Eye Study, retinal vessel diameter was associated with the prevalence of higher body mass index (BMI) and the increased risk of incident obesity [14].

In the eye, the choroid, the posterior portion of the uveal tract, nourishes the outer portion of the retina. It contributes to the blood supplied to the prelaminar portion of the optic nerve [1], is an integral constituent in the functioning of the eye, and is involved in important diseases affecting the optic nerve, retinal pigment epithelium, and the retina. By using enhanced depth imaging optical coherence tomography (EDI-OCT), choroid images can be obtained and the choroidal thickness (CT) can be measured [3].

Previous studies have suggested that a higher BMI can trigger structural changes in the retinal vascular system that could provoke retinal dysfunction, as shown in aged-related macular degeneration or diabetic retinopathy. Therefore, knowledge of the thickness changes in a vascular tissue, such as the choroid, may help to evaluate the effect of obesity on the microvascular system.

The prevalence of obesity among men and women varies greatly within, and between countries, with more obesity found in women than in men. This gender disparity in obese population is exacerbated among women in developing countries. In the TURDEP study, which investigated 24,788 people >20 years old in Turkey, the prevalence of obesity in women was 29.9, and 12.9 % in men [15].

Therefore, in the present study, we hypothesized that obesity is correlated with CT changes, particularly in women. To the best of our knowledge, this is the first study evaluating CT in obese female patients.

## Methods

This prospective clinical study included the examination of the right eyes of 72 patients. In total, 68 right eyes of 68 patients were examined and served as controls. The study was conducted between 2015 and 2016 in accordance with the tenets of the Declaration of Helsinki. The trial protocol was approved by the Local Ethical Committee of the University of Kırıkkale. Registration of the trial was requested on April 27th, 2015 (decision no:10/11). All patients and control subjects voluntarily participated in the study and signed an informed consent form. The obese group was classified according to the World Health Organisation criteria; (BMI 18.5–24.9 kg/m^2^ = normal; 25.0–29.9 kg/m^2^ = pre-obese/overweight, and ≥30.0 kg/m^2^ = obese).

In the study, the obese group included patients who had a BMI > 30 kg/m^2^, without any other disease, whereas healthy adults with BMI <25 kg/m^2^ constituted the control group. Obese patients were randomly selected from those monitored by the Department of Endocrinology. The exclusion criteria were as follows: a previous systemic or chronic disease such as hypertension, smoking, ocular surgery in one or both eyes; axial length >24 ± 1.0 mm; and a refractive measurement > 2.0 diopters.

All participants underwent a complete ocular examination, including a best-corrected visual acuity measurement, slit-lamp examination, intraocular pressure measurement, and dilated fundoscopy. Only the right eyes of each of the patients were selected to avoid any intra-individual bias.

The CT was measured as close to noon as possible to avoid diurnal variations. The measurements were performed using anEDI-OCT scanning system (OCT Advance Nidek RS-3000; Nidek Co. Ltd., Gamagori, Japan). Prior to evaluation using EDI-OCT scanning, the central macular thickness was measured in the right eye of each patient. Choroidal and scleral boundaries were drawn with the assistance of software programs. The boundaries limited the Bruch membrane, between the subfoveal points (FCT), to 500 and 1500 μm in the nasal regions (N500, N1500) and 500 and 1500 μm in the temporal regions (T500, T1500), for CT measurements. All measurements including the demarcation of the choroid and sclera were made by two independent (masked) observers. There were no significant differences between the results of the two observers (*p* = 0.317: Paired *t*-test, *r* = 0.716 and *p*˂0.001:Pearson’s correlation), and the average of the two results was used in our analyses.

Statistical analyses were performed using the SSPS statistical software (SPSS for Windows 23.0, Inc., Chicago, USA). The results of the descriptive analysis were provided in numbers, percentages, mean, median, and standard deviations. A paired *t*-test was used to assess the difference in the means of the observers’ measurements to test the repeatability and accuracy of the two independent measurements. The independent *t*-test was used to compare the variables between the obese group and the control group, and correlations were performed using Pearson’s correlation coefficient. A multiple linear regression analysis (forward) was used to determine confounding factors among the variables. *p* < 0.05 was considered statistically significant.

## Results

The study group consisted of 140 female (100 %) subjects, with a mean age of 37.55 ± 1.01 years (median:38; range:21–59 years). There were 72 patients in the obese group, with a mean age of 37.27 ± 1.18 years (median: 38.5; range 21–59 years). The control group included 68 subjects, with a mean age of 37.85 ± 7.98 years (median:38; range 24–54 years). There was no significant difference, in terms of age, between the two groups (*p* > 0.5). Demographics of the study groups are shown in Table 1.

**Table 1:** Demographics of the groups

Group	Age	BMI (Kg/m^2^)	Weight (kg)	Height (cm)
1(*N* = 72)	37.27 ± 11.81	39.16 ± 6.88	100.63 ± 16.49	160.42 ± 6.20
2(*N* = 68)	37,85 ± 7.98 *P* =0.738	21.95 ± 59 *P* < 0.001	58.97 ± 16.49 *P* =0.004	168.36 ± 4.94 *P* < 0.001

*BMI* body mass index

There was no significant difference found for foveal retinal thickness (FT) when the two groups were compared (*p* > 0.5). In contrast, the CT revealed significant differences at FCT, T500, N500, and N1500 between the two groups (all *p* < 0.05). Changes in both FT and CT are demonstrated in Table 2.

**Table 2:** Changes in foveal thickness and choroidal thickness

Group	Group	FCT	T500	T1500	N500	N1500
1(*N* = 72)	250.1 ± 19.8	349.2 ± 58.7	346.5 ± 55.1	335.8 ± 55.1	345.5 ± 59.7	327.0 ± 55.3
2(*N* = 68)	256.0 ± 18.9	322.7 ± 37.8	317.3 ± 39.7	313.6 ± 39.7	323.3 ± 39.5	322.2 ± 37.7
	*P* =0.185	*P* =0.002	*P* =0.014	*P* =0.230	*P* <0.001	*P* <0.001

FCT: choroidal thickness at fovea; N500, choroidal thickness at 500 μm nasal to the fovea; N1500, choroidal thickness at 1500 μm nasal to the fovea; T500, choroidal thickness at 500 μm temporal to the fovea; T1500, choroidal thickness at 1500 μm temporal to the fovea; FT: central macular thickness

There was a positive correlation found between BMI and CT at FCT, T500, and N500 (Table 3). Multiple linear regression analysis revealed that CT had been affected by BMI independently from the aspect of age of the patient (Table 4).

**Table 3:** The Pearson Correlation analysis between body mass index - Choroidal thickness and foveal thickness

AGE	FCT	N500	N1500	T500	T1500	FT
*r* = 0.125	*r* = 0.198	*r* = 0.193	*r* = 0.082	*r* = 0.206	*r* = 0.151	*r* = −0.162
*P* =0.140	*P* =0.019	*P* =0.022	*P* =0.334	*P* =0.015	*P* =0.076	*P* =0.056

FCT: choroidal thickness at fovea; N500, choroidal thickness at 500 μm nasal to the fovea; N1500, choroidal thickness at 1500 μm nasal to the fovea; T500, choroidal thickness at 500 μm temporal to the fovea; T1500, choroidal thickness at 1500 μm temporal to fovea; FT: central macular thickness

**Table 4:** Multiple linear regression analysis between choroidal thickness, age and Body mass index

		Beta (β)	*P*
N500	BMI	0.233	0.004
	Age	−0.317	<0.001
N1500	BMI	0.249	0.011
	Age	−0.325	0.012
T500	BMI	−0.402	0.001
	Age	−0.289	<0.001
T1500	BMI	0.204	0.009
	age	−0.423	<0.001
FCT	BMI	0.243	0.002
	Age	−0.364	<0.001

FCT: choroidal thickness at fovea; N500, choroidal thickness at 500 μm nasal to the fovea; N1500, choroidal thickness at 1500 μm nasal to the fovea; T500, choroidal thickness at 500 μm temporal to the fovea; T1500, choroidal thickness at 1500 μm temporal to the fovea; BMI: Body mass index

## Discussion

In the eye, CT may be affected by several factors, such as age, axial length, and refractive errors [16, 17]. Diurnal changes in CT have also been reported [18]. It is believed that systemic blood pressure and intraocular pressure induce choroidal tissue changes through an autoregulatory mechanism [19]. Therefore, because the choroid possesses a rich vascular structure, all of the aforementioned factors have the potential to alter the CT [20].

A study by Tanabe et al., demonstrated a significant correlation between choroidal vein diameter and the CT [21]. Another investigation by Vance et al., reported that phosphodiesterase-5 inhibitors, such as sildenafil citrate, increased CT via a smooth muscle relaxation effect [22]. In a study by Wong et al., CT was found to be thicker in hypercholesterolemic patients [23]. This study had a cross-sectional design with only Chinese subjects; therefore, their results may not address the issue of any ethnic differences in CT. It is of interest that Regatieri et al., found that the choroid was thinner among subjects with diabetic retinopathy [24]. However, a previously observed inverse correlation between age and CT might have affected this correlation [25]. A number of studies have found that CT plays a prognostic or predictive role in various local (for example, diabetic retinopathy, and AMD), and systemic diseases (for example, hypertension, anemia, and rheumatoid arthritis) [24, 26–33].

Jongh et al., reported the effects of obesity on the microvascular system; hyperinsulinemia and elevated blood pressure were found to be the major causes of the vascular alterations in obese women [4]. In another study by Kawasaki et al., both retinal venous and arterial dilatation were found in hypertensive patients [7]. Research by Saito et al., studied the retinal venous system in 900 subjects and reported an incidence of 5 years of obesity in some patients [34]. The authors found a positive correlation between vessel caliber and BMI; however, no correlation was shown between these changes and the development of obesity.

In this study, CT was found to be significantly reduced in the non-obese controls, except for the temporal measurement of 1500 μm. It was an interesting finding because as shown in recent studies, we expected a subfoveal or temporal change in CT. Previous studies reported that the macula demonstrated a thin choroid layer in the nasal region [35, 36]. Another possibility for this regional difference may be a result of the developmental pattern of the eye.

In the light of previous reports, we hypothesized that there is a relationship between obesity and the choroidal layer of the eye. In the present study, the obesity group consisted of patients with a BMI > 30, and subjects with a BMI < 25 constituted the control group. To avoid any diurnal effect, we performed all the measurements at noon for each patient. We also excluded patients with a history of local and systemic diseases. Although no significant differences were found for FT between the groups, there was a significant increase in CT at certain points (CFT, nasal 500, and 1500 μm, and temporal 500 μm) in the obese group. The results indicated that there was a positive correlation between BMI and CT, and multiple linear regression analysis revealed that CT was independently affected by the age of the patients.

There were some limitations in the study, such as the pathogenesis of obesity, which included several unknown hormonal and genetic factors; moreover, because choroid is a vascular tissue, it may be affected by local and systemic factors. We also excluded patients with systemic metabolic disorders to avoid confounding factors. A further limitation of the study was the lack of data on CT changes after weight loss through dietary restriction. The prevalence of obesity among women is greater than that in men, which we found to be the same for the patients in our Department of Endocrinology. Due to the difficulty in making homogenous groups of obese patients of both genders and the fact that choroidal tissue may be different in both genders, we decided to include only females in our study. Indeed, it may be proposed that obese male patients also have choroidal changes; therefore, further studies with male patients are warranted in the future.

## Conclusion

In summary, our data provides evidence for a relationship between CT and obesity in female patients. Vascular abnormalities may occur at early stages in obesity and ocular circulation may be a preferred target for the disease process. The assessment of CT is a quick and non-invasive technique, which can be utilized to determine such abnormalities. Meanwhile, it is unclear how this data may be applied to individual patients and how it can benefit obesity management. The data suggests that CT measurement has a predictive role and BMI should be included among the parameters that may affect CT results in obese women. A prospective follow-up study with a large sample size is required to test our hypothesis and to verify the results of the present clinical study.

## Ethics approval and consent to participate

The study protocol was approved by the Institutional Review Board (Local Ethical Committee of the Kırıkkale University), and informed written consent was obtained from all participants. The design of the study followed the tenets of the Declaration of Helsinki for biomedical research.

## Consent to publish

All authors have given consent to the publication for this manuscript.

## Availability of data and materials

In this study data supporting findings can be found in Kırıkkale University, School of Medicine, Department of Ophthalmology, Dr Erhan Yumusak; Mail: Erhanyumusak@yahoo.com.

## Abbrevations

BMI: body mass index
CT: choroidal thickness
EDI-OCT: enhanced-depth imaging optical coherence tomography
FCT: choroidal thickness at fovea
FT: foveal thickness (Central macular thickness)
N1500: choroidal thickness at 1500 μm nasal to the fovea
N500: choroidal thickness at 500 μm nasal to the fovea
RPE: retinal pigment epithelium
T1500: choroidal thickness at 1500 μm temporal to the fovea
T500: choroidal thickness at 500 μm temporal to the fovea
